# Supplementary material for: Murine hematopoietic stem cell activity is derived from pre-circulation embryos but not yolk sacs
Source: Nat Commun. 2018 Dec 20;9:5405. doi: 10.1038/s41467-018-07769-8 (PMC6302089; doi:10.1038/s41467-018-07769-8)
Supplement: Supplementary file 1 — Supplementary Information [file 41467_2018_7769_MOESM1_ESM.pdf]

Supplementary Information

Murine hematopoietic stem cell activity is derived from pre-circulation embryos but not yolk sacs.

Ganuza *et al.*

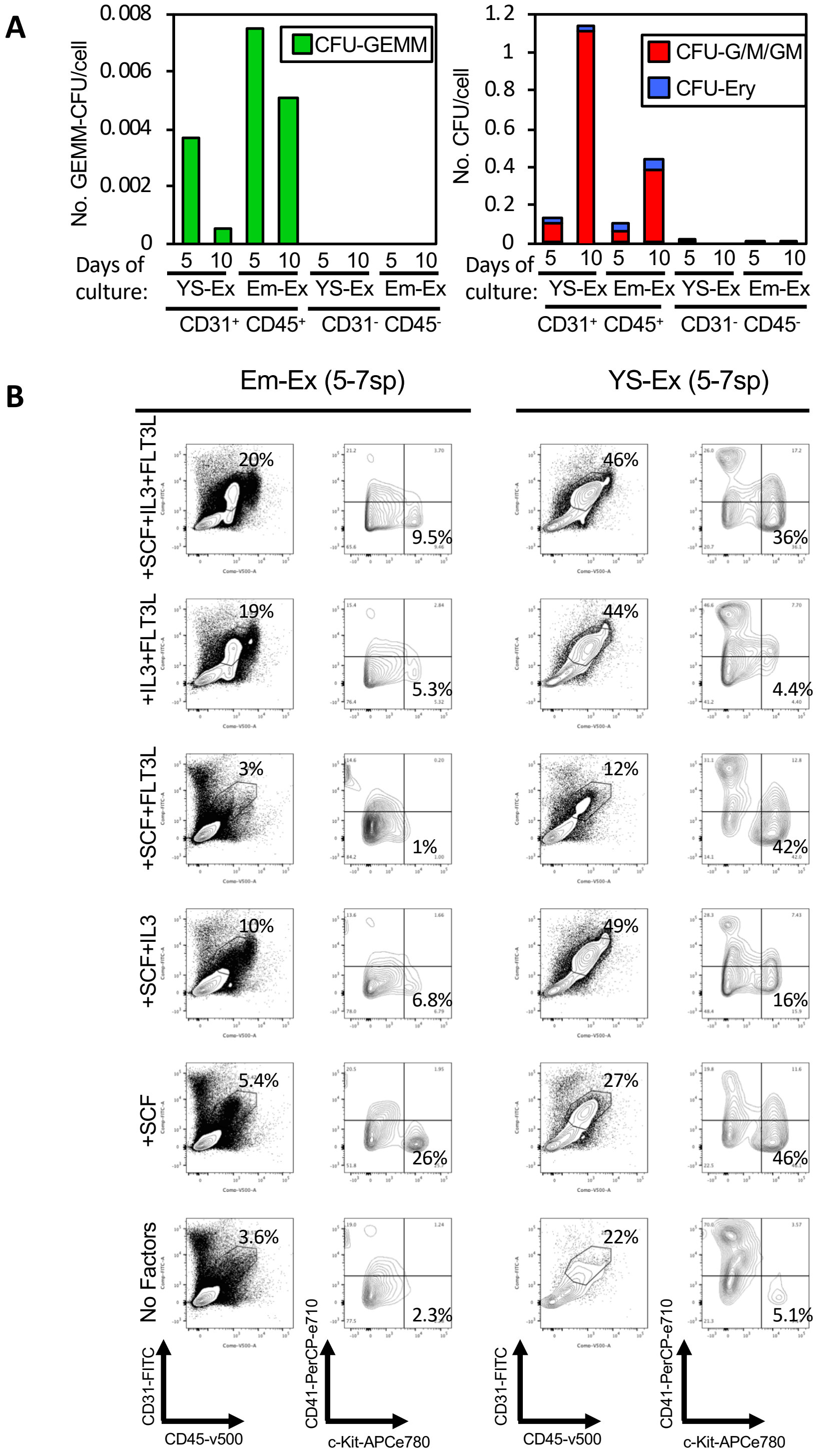

Supplementary Figure 1. CFU potential of YS-Ex and Em-Ex and flow cytometry plots related to Figure 3E. A) CD31+CD45+ and CD31^{-}CD45^{-} cells were isolated by FACS from E8.5 (5-7sp) YS-Ex and Em-Ex cultured for five or 10 days and then plated into semi-solid media supplemented with hematopoiesis promoting cytokines. The numbers of CFUs were scored 10 days later. Cumulative data (not mean) from n=4 and n=3 independent experiments is shown respectively for the five and 10 days culture periods. In particular the ratio between the total number of colonies obtained over the course of the different experiments and the total number of cells plated over all of the experiments is shown. Left panel: CFU activity per cell plated is indicated for CD31^{+}CD45^{+} and CD31^{-}CD45^{-} cells isolated after five or 10 days of explant culture. Right panel: The number of CFU-GEMM per cell is shown for CD31^{+}CD45^{+} and CD31^{-}CD45^{-} cells isolated and plated after five or 10 days of explant culture. **B)** Representative flow cytometry plots for CD31^{+}CD45^{+}c-Kit^{+}CD41^{-} cells emerging when E8.5 (5-7sp) YS-Ex and Em-Ex were cultured in the presence of different combinations of SCF, IL-3 and FLT3L. Plots correspond to the data presented in Figure 3E. Source data are provided as a Source Data file.

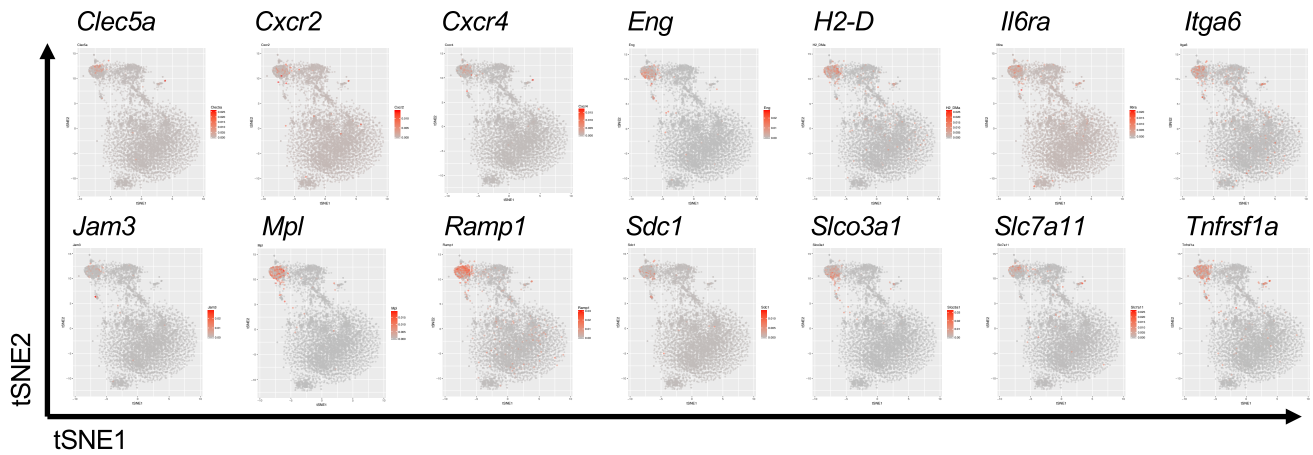

Supplementary Figure 2. Analysis of differentially expressed surface markers in X-cells. Related to Figure 4C and 4E. Single cell gene expression analysis of Em-Ex- and YS-Ex-CD31+ CD45+c-Kit+CD41- cells are shown (n=3504 Em-Ex cells; n=3037 YS-Ex cells). In particular, expression of cell surface markers enriched in X-population are depicted. Projection of single cell gene expression profiles onto tSNE1 (X-axis) vs. tSNE2 (Y-axis) are indicated Color scale indicates gene expression levels.

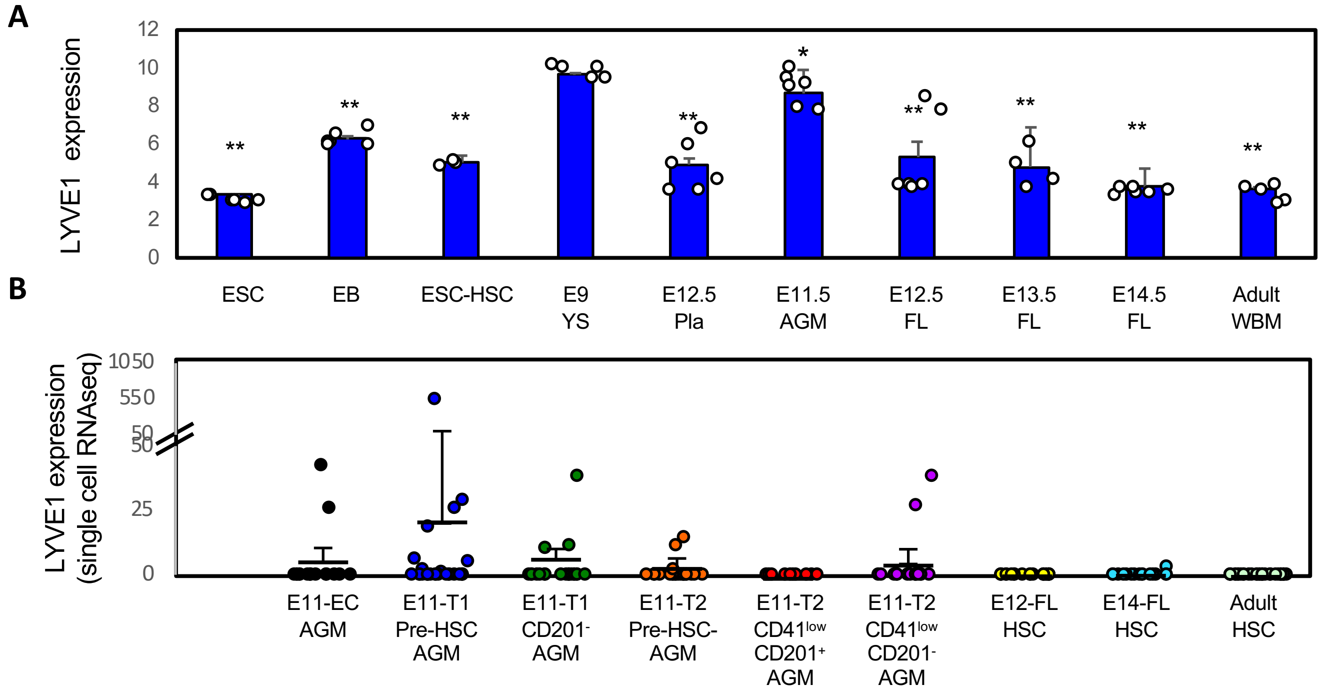

Supplementary Figure 3. *Lyve1* mRNA expression in hematopoietic precursors and stem and progenitors during ontogeny. A) Expression levels of *Lyve1* mRNA extracted from the Stemsite portal (http://daleystem.hms.harvard.edu/)[1], E9-YS (E9-CD41^{+}c-Kit^{+}CD34^{+} YS cells; n=5), E11.5-AGM (E11.5 VE-Cadherin^{+}CD45^{+} AGM cells, n=6), E12.5-Pla (E12.5-CD45^{+}c-Kit^{+}CD34^{med} placenta Cells; n=6), E12.5-FL (E12.5 Lin^{-}Sca-1^{+}c-Kit^{+}VE-Cadherin^{+}Mac1^{low} FL cells; n=6), E13.5-FL (E13.5 Lin^{-}Sca-1^{+}c-Kit^{+}CD150^{+}CD48^{-} FL cells; n=4), E14.5-FL (E14.5 Lin^{-}Sca-1^{+}c-Kit^{+}CD150^{+}CD48^{-} FL cells; n=6), Adult WBM (Lineage^{-}Sca-1^{+}c-Kit^{+}CD150+CD48- adult bone marrow cells; n=5), ESC (embryonic stem cells; n=6), EB (CD41^{+}c-Kit^{+} cells isolated from day 6 murine embryoid bodies; n=6), ESC-HSC (CD41^{bright}CD45^{-}CD34^{-} cells; n=4)[2]. Both E9-YS and E11.5-AGM showed statistically significantly higher levels of *Lyve1* mRNA than any of the other analyzed populations (**: $p < 0.01$). E9-YS also showed higher levels than E11.5-AGM (*: $p < 0.05$, two sample t-test and eWrs-test). In most cases, differences held even after multiple testing corrections to control a FDR of 0.05. Only the statistical significant difference between E9-YS and E11.5-AGM did not pass FDR control of 0.05. **B)** Expression levels of *Lyve1* mRNA from single-cell RNA-seq data. This data was extracted from the analysis of the data in Supplementary Fig. 3 from [3]. Analysis shows *Lyve1* mRNA expression levels from different populations throughout HSC ontogeny: in the E11 AGM region E11-EC-AGM (E11-CD31^{+}VE-cadherin^{+}CD41^{-}CD43^{-}CD45^{-}Ter119^{-} -AGM cells; n=17); E11-T1 pre-HSCs-AGM (E11-CD31^{+}CD45^{-}CD41^{low}c-Kit^{+} CD201^{high}-AGM cells; n=27); E11-T2 pre-HSCs-AGM (CD31^{+}CD45^{+}c-Kit^{+} CD201^{high} cells; n=22); E11-T2 CD41^{low}CD201^{+} AGM (E11-CD31^{+}CD45^{+}CD41^{low}CD201^{+}-AGM cells; n=9); E11-T2 CD41^{low}CD201^{-} AGM (E11-CD31^{+}CD45^{+}CD41^{low}CD201^{-} -AGM cells; n=16); E12-FL-HSCs, (E12-Lin^{-}Sca-1^{+}Mac-1^{low}CD201^{+}-FL cells; n=22); E14-FL-HSCs (E14-CD45^{+}CD150^{+}CD48^{-}CD201^{+}-FL cells; n=16) and Adult HSCs (CD150^{+}CD48^{-} Lineage^{-}Sca-1^{+}c-Kit^{+} cells; n= 31). Means and standard deviations are indicated. Source data are provided as a Source Data file.

Supplementary References

1 McKinney-Freeman, S. *et al.* The transcriptional landscape of hematopoietic stem cell ontogeny. *Cell Stem Cell* **11**, 701-714, doi:10.1016/j.stem.2012.07.018 (2012).

2 McKinney-Freeman, S. L. *et al.* Surface antigen phenotypes of hematopoietic stem cells from embryos and murine embryonic stem cells. *Blood* **114**, 268-278, doi:10.1182/blood-2008-12-193888 (2009).

3 Zhou, F. *et al.* Tracing haematopoietic stem cell formation at single-cell resolution. *Nature* **533**, 487-492, doi:10.1038/nature17997 (2016).
