# Supplementary material for: Murine hematopoietic stem cell activity is derived from pre-circulation embryos but not yolk sacs
Source: Nat Commun. 2018 Dec 20;9:5405. doi: 10.1038/s41467-018-07769-8 (PMC6302089; doi:10.1038/s41467-018-07769-8)
Supplement: Supplementary file 3 — Description of Additional Supplementary Files [file 41467_2018_7769_MOESM3_ESM.docx]

**Title:** Source Data
**Description:** underlying Figs. 1, 2, 3, 4A, 5, 6A-C and 7 and Supplementary Figs. 1A, and 3 are provided as a Source Data file
